# Synthesis and Antimycobacterial and Photosynthesis-Inhibiting Evaluation of 2-[(*E*)-2-Substituted-ethenyl]-1,3-benzoxazoles

**DOI:** 10.1155/2014/705973

**Published:** 2014-08-13

**Authors:** Ales Imramovsky, Jan Kozic, Matus Pesko, Jirina Stolarikova, Jarmila Vinsova, Katarina Kralova, Josef Jampilek

**Affiliations:** ^1^Institute of Organic Chemistry and Technology, Faculty of Chemical Technology, University of Pardubice, Studentska 573, 532 10 Pardubice, Czech Republic; ^2^Department of Inorganic and Organic Chemistry, Faculty of Pharmacy in Hradec Kralove, Charles University in Prague, Heyrovskeho 1203, 500 05 Hradec Kralove, Czech Republic; ^3^Department of Environmental Ecology, Faculty of Natural Sciences, Comenius University, Mlynska dolina Ch-2, 842 15 Bratislava, Slovakia; ^4^Laboratory for Mycobacterial Diagnostics and TB, Institute of Public Health in Ostrava, Partyzanske namesti 7, 702 00 Ostrava, Czech Republic; ^5^Institute of Chemistry, Faculty of Natural Sciences, Comenius University, Mlynska dolina Ch-2, 842 15 Bratislava, Slovakia; ^6^Department of Chemical Drugs, Faculty of Pharmacy, University of Veterinary and Pharmaceutical Sciences Brno, Palackeho 1/3, 612 42 Brno, Czech Republic

## Abstract

A series of twelve 2-[(*E*)-2-substituted-ethenyl]-1,3-benzoxazoles was designed. All the synthesized compounds were tested against three mycobacterial strains. The compounds were also evaluated for their ability to inhibit photosynthetic electron transport (PET) in spinach (*Spinacia oleracea* L.) chloroplasts. 2-[(*E*)-2-(4-Methoxyphenyl)ethenyl]-1,3-benzoxazole, 2-[(*E*)-2-(2,3-dihydro-1-benzofuran-5-yl)ethenyl]-1,3-benzoxazole and 2-{(*E*)-2-[4-(methylsulfanyl)phenyl]ethenyl}-1,3-benzoxazole showed the highest activity against *M. tuberculosis*, *M. kansasii,* and *M. avium*, and they demonstrated significantly higher activity against *M. avium* and *M. kansasii* than isoniazid. The PET-inhibiting activity of the most active *ortho*-substituted compound 2-[(*E*)-2-(2-methoxyphenyl)ethenyl]-1,3-benzoxazole was IC_50_ = 76.3 *μ*mol/L, while the PET-inhibiting activity of *para*-substituted compounds was significantly lower. The site of inhibitory action of tested compounds is situated on the donor side of photosystem II. The structure-activity relationships are discussed.

## 1. Introduction

The design of new compounds that focused on resistant bacteria, mycobacteria, and fungi has become one of the most important areas of antimicrobial research today, since resistance of pathogenic bacteria toward available antimicrobial drugs is rapidly becoming a major problem worldwide. The increasing incidences of tuberculosis (TB), the number of cases of multi-drug-resistant strains of* Mycobacterium tuberculosis* (MDR-TB), and infections by nontuberculous mycobacteria (NTM) that are connected with the increase of the number of immunocompromised patients and evolving resistance mycobacterial species to antimycobacterial chemotherapeutics make the discovery of new molecular scaffolds a priority [1–5]. Because of the high profile of* M. tuberculosis,* the pathogenic role of other NTM in humans was overshadowed for a long time. For example,* M. kansasii* (one of the most virulent NTM) causes always more common nontuberculous mycobacterial lung infections [6]; therefore, it is urgent to search novel potential antimycobacterial agents not only against TB/MDR-TB but also against NTM strains.

Benzoxazole and its isosteres benzimidazole and benzothiazole represent privileged structures, that is, they are useful ligands for more than one type of receptor or enzyme targets by judicious structural modifications. They are privileged structural units not only in the pharmaceutical industry but also in several other fields such as agricultural, electronic, and polymer chemistry. The benzoxazole system is present in numerous antimicrobial agents [5, 7–15]. It was also found that benzoxazoles and benzothiazoles exhibit significant herbicidal [16–25] and antialgal activity [18, 19, 26, 27].

Benzoxazoles can be considered as structural bioisosteres of nucleotides such as adenine and guanine, which allow them to interact easily with the biopolymers of a living system [10, 28]. Also it was found that benzoxazoles inhibit essential bacterial enzymes, such as hyaluronan lyase [11] and isocitrate lyase [12], as well as bacterial two-component systems [29–31]. Although the mode of action of some herbicidal benzoxazoles and benzothiazoles consists in inhibition of fatty acid synthesis, over 50% of commercially available herbicides act by reversible binding to photosystem II (PS II), a membrane-protein complex in the thylakoid membranes, which catalyses the oxidation of water and the reduction of plastoquinone [32], and thereby inhibit photosynthesis [33–35].

Both pharmaceuticals and pesticides (including herbicides) are designed to target particular biological functions, and in many cases they target similar processes or have similar molecular sites of action. For many years, virtually all pharmaceutical companies had agrochemical divisions. Leads for pharmaceuticals and pesticides often overlap, in some cases leading to similar compounds used for human health and weed management purposes. Multiple activities of various herbicides/herbicide classes show potential pharmaceutical properties, both as therapeutic agents that act through human molecular target sites and those that act on infectious agents [36–38]. Moreover, good correlation between antimicrobial activities and herbicidal effects was found [19, 25, 39–45].

The prepared 2-styrylbenzoxazole-like compounds were designed as potential antimycobacterial agents and tested against three mycobacterial species. As it is known that a number of antimicrobial compounds display also photosynthesis inhibiting activity (bond to PS II) [19, 25, 39–44], all the prepared compounds were evaluated in relation to inhibition of photosynthetic electron transport (PET) in spinach (*Spinacia oleracea* L.) chloroplasts. Relationships between the structure and* in vitro* antimycobacterial activities or/and inhibitory activity related to inhibition of photosynthetic electron transport (PET) in spinach chloroplasts of the new compounds are discussed.

## 2. Material and Methods

### 2.1. Chemistry

The chemicals were purchased from commercial sources (Sigma Aldrich, Acros Organics, TCI, Merck). Commercial grade reagents were used without further purification. Reactions were monitored by means of thin layer chromatography plates coated with 0.2 mm Silica Gel 60 F254 (Merck). TLC plates were visualized by UV irradiation (254 nm). The products were purified by crystallization, by means of column chromatography employing Silica Gel 60 (Merck). All melting points were determined on Melting Point B-540 apparatus (Buchi, Germany) and are uncorrected. NMR spectra were measured in CDCl_3_ solutions on a Bruker Avance 300 or 500 MHz apparatus. The chemical shifts *δ* are given in ppm, relating to chemical shift of the solvent. The coupling constants (*J*) are reported in hertz (Hz). Elemental analysis (C, H, N) was performed on a Thermo Scientific Flash 2000 Organic elemental analyser.

#### 2.1.1. Synthesis

General procedure for synthesis of* 2-[(E)-2-substituted-ethenyl]-1,3-benzoxazoles (* ***1*** *–* ***14***): 2-Methylbenzo[*d*]oxazole (6.78 mmol) and the appropriate aldehyde (1.695 mmol) were added to a stirred mixture of dry THF (10 mL) and* t*-BuOH (2 mL) under argon atmosphere. Then the mixture was cooled to −50°C and 1 M solution of potassium* tert*-butoxide in THF (2 mL) was slowly added to keep temperature below −50°C. During addition, the reaction mixture changed light yellow colour to green. Reaction mixture was stirred at −50°C for 2 hours. Temperature was then raised to approx. −26°C and kept for 3 hours. After that the reaction mixture was kept at ambient temperature and dark for 18 hours. The reaction mixture was then treated with 5% water solution of sodium hydrogen carbonate (30 mL) and toluene (30 mL). The organic phase was extracted with brine, dried over Na_2_SO_4,_ and filtered, and the solvent was evaporated under reduced pressure. The mixture contained the desired product, and starting 2-methylbenzo[*d*]oxazole was purified by column chromatography (hexane-ethyl acetate, 9 : 1). Given are yields of isolated compounds.

*2-[(E)-2-Phenylethenyl]-1,3-benzoxazole (* ***1***). White solid. Yield 41%; mp 81.6–82.5°C (86–88°C [46]). IR (KBr, cm^−1^): 3062, 3040, 2360, 2343, 1642, 1535, 1454, 1350, 1237, 1178, 1108, 1004, 967, 933, 863, 840, 764, 743, 7014, 684, 497, 434. ^1^H NMR (300 MHz, CDCl_3_), *δ*: 7.80 (1H, d,* J* = 16.2 Hz), 7.72 (1H, m), 7.60 (2H, m), 7.53 (1H, m), 7.41 (3H, m), 7.34 (2H, m), 7.09 (1H, d,* J* = 16.2 Hz). ^13^C NMR (CDCl_3_, 75 MHz), *δ*: 162.8, 150.4, 142.2, 139.4, 135.1, 129.8, 129.0, 127.5, 125.2, 124.5, 119.8, 113.9, 110.3. Anal. Calcd. for C_15_H_11_NO (221.25): C, 81.43; H, 5.01; N, 6.33; O, 7.23. Found: C, 81.60; H, 5.37; N, 6.37.

*2-[(E)-2-(2-Methoxyphenyl)ethenyl]-1,3-benzoxazole (* ***2*** *) [47]*. White solid. Yield 14%; mp. 67.0–68.2°C. IR (KBr, cm^−1^): 3061, 3001, 2949, 2842, 1924, 1884, 1811, 1637, 1596, 1577, 1545, 1532, 1485, 1453, 1468, 1438, 1345, 1320, 1305, 1286, 1249, 1203, 1183, 1163, 1103, 1053, 1025, 1003, 971, 932, 894, 866, 855, 844, 786, 760, 748, 626, 603, 576, 552, 509, 478, 428. ^1^H NMR (500 MHz, CDCl_3_), *δ*: 8.11 (1H, d,* J* = 15 Hz), 7.11 (1H, m), 7.59 (1H, dd,* J* = 10 Hz,* J* = 5 Hz), 7.53 (1H, m), 7.33 (3H, m), 7.23 (1H, d,* J* = 20 Hz), 6.98 (1H, t,* J* = 5 Hz), 6.94 (1H, d,* J* = 10 Hz), 3.93 (3H, s). ^13^C NMR (125 MHz, CDCl_3_), *δ*: 163.5, 158.14, 150.3, 141.6, 135.6, 131.0, 128.4, 125.2, 124.0, 121.5, 119.6, 114.2, 11.2, 110.4, 55.5. Anal. Calcd. for C_16_H_13_NO_2_ (251.28): C, 76.48; H, 5.21; N, 5.57; O, 12.73. Found: C, 76.36; H, 5.37; N, 5.74.

*2-[(E)-2-(4-Methoxyphenyl)ethenyl]-1,3-benzoxazole (* ***3***). Yellowish solid. Yield 56%; mp. 133.2–134.7°C (138-139°C [48]). IR (KBr, cm^−1^): 3403, 3047, 2973, 2926, 2839, 1886, 1774, 1642, 1600, 1537, 1506, 1455, 1350, 1254, 1207, 1172, 1145, 1117, 1030, 1007, 962, 933, 893, 823, 760, 740, 730, 721, 573, 534, 515, 439. ^1^H NMR (500 MHz, CDCl_3_), *δ*: 7.75 (1H, d,* J* = 16.4 Hz), 7.71 (1H, m), 7.53 (3H, m), 7.32 (2H, m), 6.94 (3H, m), 3.84 (3H, s). ^13^C NMR (125 MHz, CDCl_3_), *δ*: 163.2, 161.0, 150.3, 142.2, 139.1, 129.1, 127.9, 124.9, 124.4, 119.6, 114.4, 111.5, 110.2, 55.35. Anal. Calcd. for C_16_H_13_NO_2_ (251.28): C, 76.48; H, 5.21; N, 5.57; O, 12.73. Found: C, 76.42; H, 5.30; N, 5.66.

*2-*{*(E)-2-[4-(Methylsulfanyl)phenyl]ethenyl*}*-1,3-benzoxazole (* ***4***). Yellowish solid. Yield 15%; mp. 143.0–144.1°C. IR (KBr, cm^−1^): 3446, 3064, 2920, 1639, 1590, 1556, 1529, 1491, 1472, 1453, 1435, 1407, 1349, 1305, 1290, 1244, 1177, 1119, 1099, 1088, 1053, 1025, 1002, 972, 931, 894, 865, 843, 812, 760, 747, 724, 622, 530, 507, 436. ^1^H NMR (500 MHz, CDCl_3_), *δ*: 7.83 (1H, d,* J* = 15 Hz), 7.78 (1H, m), 7.58 (3H, m), 7.40 (2H, m), 7.33 (2H, m), 7.10 (1H, d,* J* = 16.8 Hz), 2.58 (3H, s). ^13^C NMR (125 MHz, CDCl_3_), *δ*: 163.0, 150.3, 141.8, 141.4, 139.2, 131.6, 128.0, 126.1, 125.2, 124.6, 119.7, 112.6, 110.3, 15.2. Anal. Calcd. for C_16_H_13_NOS (267.35): C, 71.88; H, 4.90; N, 5.24; O, 5.98; S, 11.99. Found: C, 72.20; H, 4.98; N, 5.73.

*2-[(E)-2-(4-Methylphenyl)ethenyl]-1,3-benzoxazole (* ***5***). Yellowish solid. Yield 25%; mp. 130.3–131.0°C (130–132°C [48]). IR (KBr, cm^−1^): 3446, 3051, 3019, 2917, 1771, 1639, 1606, 1571, 1534, 1508, 1474, 1455, 1415, 1350, 1291, 1308, 1260, 1243, 1197, 1178, 1157, 1145, 1118, 1004, 976, 929, 894, 879, 865, 846, 808, 777, 760, 740, 721, 624, 532, 486, 436. ^1^H NMR (500 MHz, CDCl_3_), *δ*: 7.79 (1H, d,* J* = 16.4 Hz), 7.73 (1H, m), 7.52 (3H, m), 7.35 (2H, m), 7.25 (2H, m), 7.05 (1H, d,* J* = 16.3 Hz), 2.41 (3H, s). ^13^C NMR (125 MHz, CDCl_3_), *δ*: 163.0, 150.4, 142.2, 140.1, 139.5, 132.4, 129.7, 125.4, 119.7, 112.9, 110.2, 21.41. Anal. Calcd. for C_16_H_13_NO (235.28): C, 81.68; H, 5.57; N, 5.95; O, 6.80. Found: C, 81.70; H, 5.70; N, 6.08.

*2-[(E)-2-(4-Chlorophenyl)ethenyl]-1,3-benzoxazole (* ***6***). Yellowish solid. Yield 43%; mp. 144.0-145.0°C (148–150°C [48]). IR (KBr, cm^−1^): 3404, 3061, 1773, 1698, 1864, 1643, 1594, 1570, 1530, 1508, 1490, 1474, 1453, 1407, 1385, 1349, 1304, 1289, 1189, 1177, 1156, 1106, 1089, 1013, 1003, 969, 930, 893, 843, 813, 760, 739, 721, 669, 622, 531, 503, 435. ^1^H NMR (500 MHz, CDCl_3_), *δ*: 7.74 (2H, m), 7.54 (3H, m), 7.38 (4H, m), 7.05 (1H, d,* J* = 16.4 Hz). ^13^C NMR (125 MHz, CDCl_3_), *δ*: 162.4, 150.3, 142.0, 138.0, 135.6, 133.6, 129.2, 128.7, 125.4, 124.6, 119.9, 114.4, 110.3. Anal. Calcd. for C_15_H_10_ClNO (255.699): C, 70.46; H, 3.94; Cl, 13.87; N, 5.48; O, 6.26. Found: C, 70.30; H, 4.25; N, 5.73.

*2-*{*(E)-2-[4-(Trifluoromethyl)phenyl]ethenyl*}*-1,3-benzoxazole (* ***7***). White solid. Yield 53%; mp. 152.0–152.5°C. IR (KBr, cm^−1^): 3049, 1939, 1781, 1683, 1645, 1614, 1578, 1535, 1511, 1474, 1451, 1415, 1325, 1245, 1188, 1106, 1017, 974, 957, 843, 750, 696, 639, 594, 507, 426. ^1^H NMR (CDCl_3_, 300 MHz), *δ*: 7.80 (1H, d,* J* = 16.5 Hz), 7.76 (5H, m), 7.54 (1H, m), 7.36 (2H, m), 7.15 (1H, d,* J* = 16.5 Hz). ^13^C NMR (CDCl_3_, 75 MHz), *δ*: 162.0, 150.4, 142.0, 138.5, 137.5, 127.6, 125.9 (q,* J* = 3.675 Hz), 125.6, 124.7, 120.1, 116.4, 110.4. Anal. Calcd. for C_16_H_10_F_3_NO (289.25): C, 66.44; H, 3.48; F, 19.70; N, 4.84; O, 5.53. Found: C, 67.21; H, 3.66; N, 5.05.

*2-[(E)-2-(2,3-Dihydro-1-benzofuran-5-yl)ethenyl]-1,3-benzoxazole (* ***8***). Yellowish solid. Yield 15%; mp. 143.0-144.0°C. IR (KBr, cm^−1^): 3441, 3062, 2969, 2914, 1643, 1608, 1546, 1530, 1493, 1473, 1452, 1443, 1384, 1352, 1334, 1302, 1289, 1263, 1244, 1201, 1179, 1104, 1003, 983, 933, 893, 857, 812, 758, 737, 725, 624, 565, 529, 484, 443. ^1^H NMR: (500 MHz, CDCl_3_), *δ*: 7.77 (1H, d,* J* = 15 Hz), 7.69 (1H, m), 7.52–7.48 (1H, m), 7.48 (1H, s), 7.36 (1H, d,* J* = 10 Hz), 7.26–7.32 (2H, m), 6.92 (1H, d,* J* = 15 Hz), 6.82 (1H, d,* J* = 5 Hz), 4.63 (2H, t,* J* = 10 Hz), 3.25 (2H, t,* J* = 10 Hz). ^13^C NMR: (125 MHz, CDCl_3_), *δ*: 163.3, 161.9, 150.1, 141.5, 140.0, 129.1, 128.1, 127.8, 124.9, 124.4, 123.8, 119.3, 110.3, 110.1, 109.7, 71.7, 29.2. Anal. Calcd. for C_17_H_13_NO_2_ (263.29): C, 77.55; H, 4.98; N, 5.32; O, 12.15. Found: C, 77.27; H, 5.11; N, 5.57.

*2-[(E)-2-(Furan-2-yl)ethenyl]-1,3-benzoxazole (* ***9*** *) [49]*. Yellowish solid. Yield 36%; mp. 119.5–120.0°C. IR (KBr, cm^−1^): 3126, 3109, 1774, 1699, 1671, 1635, 1577, 1538, 1520, 1473, 1463, 1449, 1430, 1364, 1392, 1349, 1301, 1281, 1266, 1199, 1178, 1154, 1144, 1108, 1071, 1016, 1002, 957, 941, 930, 891, 882, 857, 822, 771, 758, 742, 623, 594, 527, 441, 406. ^1^H NMR (500 MHz, CDCl_3_), *δ*: 7.72 (1H, m), 7.52 (3H, m), 7.33 (2H, m), 6.96 (1H, d,* J* = 16.1 Hz), 6.62 (1H, d,* J* = 3.31 Hz), 6.51 (1H, m). ^13^C NMR (125 MHz, CDCl_3_), *δ*: 162.7, 151.4, 150.4, 144.4, 142.3, 126.0, 125.1, 124.4, 119.761, 113.5, 112.3, 111.7, 110.2. Anal. Calcd. for C_13_H_9_NO_2_ (211.20): C, 73.92; H, 4.29; N, 6.63; O, 15.15. Found: C, 74.09; H, 4.57; N, 6.77.

*2-[(E)-2-(5-Ethylfuran-2-yl)ethenyl]-1,3-benzoxazole (* ***10***). Yellowish solid. Yield 49%; mp. 78.0–78.7°C. IR (KBr, cm^−1^): 3733, 1665, 1634, 15825, 1558, 1510, 1490, 1455, 1387, 1371, 1348, 1325, 1275, 1241, 1200, 1175, 1157, 1107, 1020, 1006, 939, 923, 892, 855, 792, 774, 759, 744, 711, 643, 623, 549, 526, 441. ^1^H NMR (500 MHz, CDCl_3_), *δ*: 7.70 (1H, m), 7.49 (2H, m), 7.31 (2H, m), 6.88 (1H, d,* J* = 16.0 Hz), 6.53 (1H, d,* J* = 3.10 Hz), 6.11 (1H, m), 2.74 (2H, q,* J* = 7.71 Hz), 3.06 (3H, t,* J* = 7.7 Hz). ^13^C NMR (125 MHz, CDCl_3_), *δ*: 163.1, 160.6, 150.3, 149.9, 142.4, 126.2, 124.8, 124.3, 119.6, 114.9, 110.1, 109.9, 107.2, 21.6, 11.9. Anal. Calcd. for C_15_H_13_NO_2_ (239.20): C, 75.30; H, 5.48; N, 5.85; O, 13.37. Found: C, 75.27; H, 5.66; N, 6.02.

*2-[(1E,3E)-4-Phenylbuta-1,3-dien-1-yl]-1,3-benzoxazole (* ***11***). Yellowish solid. Yield 26%; mp. 108.5–109.0°C. IR (KBr, cm^−1^): 3056, 3022, 1876, 1774, 1630, 1621, 1596, 1525, 1488, 1471, 1448, 1553, 1304, 1283, 1243, 1206, 1179, 1138, 1126, 1104, 1072, 1027, 999, 964, 933, 893, 879, 860, 848, 831, 758, 743, 703, 690, 622, 561, 509, 443. ^1^H NMR (300 MHz, CDCl_3_), *δ*: 7.70 (1H, m), 7.57–7.47 (4H, m), 7.40–7.31 (5H, m), 6.99 (1H, d,* J* = 10.5 Hz), 6.90 (1H, d,* J* = 15.4 Hz), 6.63 (1H, d,* J* = 15.5 Hz). ^13^C NMR (75 MHz, CDCl_3_), *δ*: 162.8, 150.4, 142.3, 139.7, 138.7, 136.2, 128.8, 127.2, 127.1, 125.1, 124.5, 119.8, 120.0, 110.2. Anal. Calcd. for C_17_H_13_NO (247.29): C, 82.47; H, 5.30; N, 5.66; O, 6.47. Found: C, 82.31; H, 5.45; N, 5.81.

*2-[(1E,3E)-4-(4-Methoxyphenyl)buta-1,3-dien-1-yl]-1,3-benzoxazole (* ***12***). Yellowish solid. Yield 36%; mp. 124.5–126.0°C. IR (KBr, cm^−1^): 3437, 3013, 2841, 1631, 1593, 1574, 1524, 1510, 1473, 1454, 1440, 1422, 1384, 1352, 1303, 1283, 1262, 1244, 1203, 1188, 1177, 1150, 1030, 993, 962, 931, 893, 877, 865, 847, 838, 812, 762, 739, 636, 597, 551, 522, 440. ^1^H NMR: (500 MHz, CDCl_3_), *δ*: 7.68 (1H, m), 7.55 (1H, m), 7.47 (1H, m), 7.42 (2H, m AA′BB′), 7.30 (2H, m), 6.87 (4H, m), 6.57 (1H, d,* J* = 15 Hz), 3.81 (3H, s). ^13^C NMR: (125 MHz, CDCl_3_), *δ*: 163.0, 160.3, 150.3, 142.0, 140.5, 138.7, 129.1, 128.6, 125.1, 125.0, 124.5, 119.6, 115.5, 114.2, 110.2, 55.3. Anal. Calcd. for C_18_H_15_NO_2_ (277.32): C, 77.96; H, 5.45; N, 5.05; O, 11.54. Found: C, 77.62; H, 5.52; N, 5.20.

#### 2.1.2. Lipophilicity Determination by HPLC (Capacity Factor* k*/Calculated log* k*)

A Waters Alliance 2695 XE HPLC separation module and a Waters Photodiode Array Detector 2996 (Waters Corp., Milford, MA, USA) were used. A Symmetry C_18_ 5 *μ*m, 4.6 × 250 mm, Part No. WAT054275 (Waters Corp., Milford, MA, USA) chromatographic column was used. The HPLC separation process was monitored by Empower 2 Chromatography Data Software, Waters 2009 (Waters Corp., Milford, MA, USA). A mixture of MeOH p.a. (70%) and H_2_O-HPLC - Mili-Q Grade (30%) was used as a mobile phase. The total flow of the column was 1.0 mL/min, injection volume, 30 *μ*L, column temperature, 45°C, and sample temperature, 10°C. The detection wavelength of 210 nm was chosen. The KI methanolic solution was used for the dead time (*t*_*D*_) determination. Retention times (*t*_*R*_) were measured in minutes. The capacity factors* k* were calculated using the Empower 2 Chromatography Data Software according to formula* k* = (*t*_*R*_ − *t*_*D*_)/*t*_*D*_, where *t*_*R*_ is the retention time of the solute, whereas *t*_*D*_ denotes the dead time obtained using an unretained analyte. The log *k* values of the individual compounds are shown in Table 1.

### 2.2. Biology

#### 2.2.1. *In Vitro* Antimycobacterial Evaluation

The* in vitro* antimycobacterial activity of all prepared compounds was evaluated against* Mycobacterium tuberculosis* (MTB) CNCTC My 331/88 (identical with H37Rv and ATCC 27294, dilution of the strain was 10^−3^),* Mycobacterium avium* (MA) CNCTC My 330/88 (identical with ATCC 25291, dilution of the strain was 10^−5^), and* Mycobacterium kansasii* (MK) CNCTC My 235/80 (identical with ATCC 12478, dilution of the strain was 10^−4^) in the Laboratory for Mycobacterial Diagnostics and TB, the Institute of Public Health in Ostrava, the Czech Republic. All strains were obtained from the Czech National Collection of Type Cultures (CNCTC). Antimycobacterial activities were determined in the Sula semisynthetic medium (Sevac, Prague, Czech Republic). Each strain was simultaneously inoculated into Petri plates containing the Lowenstein-Jensen medium for the control of the inoculum sterility and growth. The tested compounds were added to the medium as DMSO solutions. The following concentrations were used: 250, 125, 62, 32, 16, 8, 4, 2, and 1 *μ*mol/L. Inoculated plates kept in microtone bags were incubated at 37°C. Reading was carried out on a stand with a bottom magnifying mirror, macroscopically, with the use of a magnifying glass. The growth in plates was evaluated after 14 and 21 days of the incubation. The growth of the colonies in a control well plate, corresponding to the growth into 100 colonies in the control Lowenstein-Jensen medium, was considered as optimum dilution for evaluation of the results. In the course of the test evaluation, the minimum inhibitory concentration (MIC (*μ*mol/L)) was considered as the lowest substance concentration at which the inhibition of mycobacteria growth occurs [50]. The MIC value is routinely and widely used in bacterial assays and is a standard detection limit according to the Clinical and Laboratory Standards Institute (CLSI). Isoniazid was used as the reference antimycobacterial drug. The results are summarized in Table 1.

#### 2.2.2. Study of Inhibition of Photosynthetic Electron Transport (PET) in Spinach Chloroplasts

Chloroplasts were prepared from spinach (*Spinacia oleracea* L.) according to Masarovicova and Kralova [51]. The inhibition of photosynthetic electron transport (PET) in spinach chloroplasts was determined spectrophotometrically (Genesys 6, Thermo Scientific), using an artificial electron acceptor 2,6-dichlorophenol-indophenol (DCPIP) according to Kralova et al. [16], and the rate of photosynthetic electron transport was monitored as a photoreduction of DCPIP. The measurements were carried out in phosphate buffer (0.02 mol/L, pH 7.2) containing sucrose (0.4 mol/L), MgCl_2_ (0.005 mol/L), and NaCl (0.015 mol/L). The chlorophyll content was 30 mg/L in these experiments and the samples were irradiated (~100 W/m^2^ with 10 cm distance) with a halogen lamp (250 W) using a 4 cm water filter to prevent warming of the samples (suspension temperature 22°C). The studied compounds were dissolved in DMSO due to their limited water solubility. The applied DMSO concentration (up to 4%) did not affect the photochemical activity in spinach chloroplasts. The inhibitory efficiency of the studied compounds was expressed by IC_50_ values, that is, by molar concentration of the compounds causing 50% decrease in the oxygen evolution rate relative to the untreated control. The comparable IC_50_ value for a selective herbicide 3-(3,4-dichlorophenyl)-1,1-dimethylurea, DCMU (Diuron), was about 1.9 *μ*mol/L. The results are shown in Table 1.

#### 2.2.3. Study of Aromatic Amino Acids Fluorescence in Spinach Chloroplasts

The fluorescence emission spectra of aromatic amino acids (AAA) in spinach chloroplasts were recorded on fluorescence spectrophotometer F-2000 (Hitachi, Tokyo, Japan) using excitation wavelength *λ*_ex_ = 275 nm, excitation slit 20 nm, and emission slit 10 nm. The phosphate buffer used for dilution of the chloroplast suspension was the same as described above. Due to low aqueous solubility the compounds were added to the chloroplast suspension in DMSO solution. The DMSO concentration in all samples was the same as in the control (10%). The chlorophyll concentration in the chloroplast suspension was 10 mg/L.

## 3. Results and Discussion

### 3.1. Chemistry

#### 3.1.1. Synthesis

**Scheme 1:**
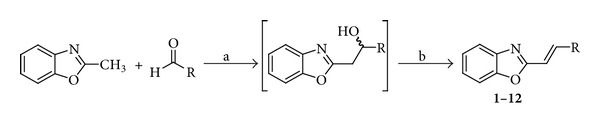
Synthesis of 2-[(*E*)-2-substituted-ethenyl]-1,3-benzoxazoles** 1**–**12**.* Reagents and conditions: *(a)* t*-BuOK, THF, −50°C; (b) ambient temperature.

2-Methylbenzo[*d*]oxazole with the appropriate aldehyde in dry THF and* t*-BuOH at −50°C provided an intermediate that under stirring and ambient temperature yielded the desired product. All 2-substituted 1,3-benzoxazoles** 1**–**12** were prepared according to Scheme 1.

#### 3.1.2. Lipophilicity

**Figure 1:**
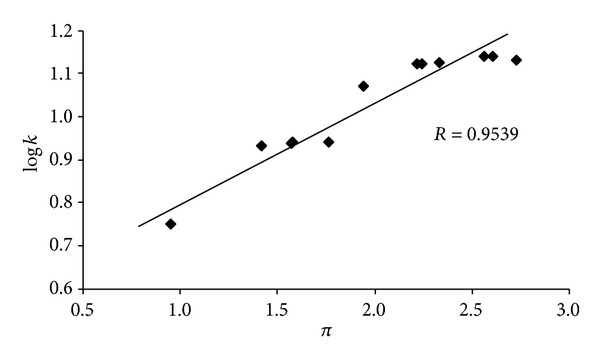
Comparison of experimentally found log* k* values with calculated distributive parameters *π* (ACD/Percepta) of substituents of 2-[(*E*)-ethenyl]-1,3-benzoxazoles** 1**–**12**.

Lipophilicity is a property that has a major effect on ADME/Tox properties as well as pharmacological activity, because drugs mostly cross biological membranes through passive transport, which strongly depends on their lipophilicity. Lipophilicity has been studied and applied as an important drug property for decades. This parameter was measured by means of RP-HPLC and expressed as logarithm of capacity factor* k*. The procedure was performed under isocratic conditions with methanol as an organic modifier in the mobile phase using an end-capped nonpolar C_18_ stationary RP column. The hydrophobic contributions (expressed as distributive parameters *π*) of individual substituents in C_(2)_ position of 2-[(*E*)-2-substituted-ethenyl]-1,3-benzoxazole scaffold were predicted using ACD/Percepta ver. 2012 (Advanced Chemistry Development, Inc., Toronto, ON, Canada). The results are shown in Table 1 and the match of the experimental determined lipophilicity log* k* with calculated distributive parameters *π* is illustrated in Figure 1.

Compounds from the series of 2-[(*E*)-2-substituted-ethenyl]-1,3-benzoxazoles showed a wide range of lipophilicities with log* k* values from ca. 0.75 (**9**) to ca. 1.14 (**11**). The results obtained for compounds** 1**–**12** show that the experimentally determined lipophilicities (log* k*) of the discussed compounds are in accordance with the calculated *π* values of the compounds as shown in Figure 1  (*n* = 12, *R* = 0.9539).

Based on the results it can be stated that heterocycles demonstrated lower lipophilicity (**9** <** 8**) than phenyl (**1**); only compound** 10** showed higher lipophilicity than** 1**. 2-[(1*E*,3*E*)-4-arylbuta-1,3-dien-1-yl]-1,3-benzoxazoles** 11** and** 12** expressed higher lipophilicity than styryl derivatives** 1**–**7**. Lipophilicity of these derivatives increased as follows: OCH_3_ < SCH_3_ < CH_3_ < Cl < CF_3_. The 2-methoxy moiety (compound** 2**) showed lower lipophilicity than the 4-methoxy moiety (compound** 3**) that demonstrated slightly lower lipophilicity than its cyclic analogue 2-[(*E*)-2-(2,3-dihydro-1-benzofuran-5-yl)ethenyl]-1,3-benzoxazole (**8**).

### 3.2. Biology

#### 3.2.1. *In Vitro* Antimycobacterial Evaluation

**Figure 2:**
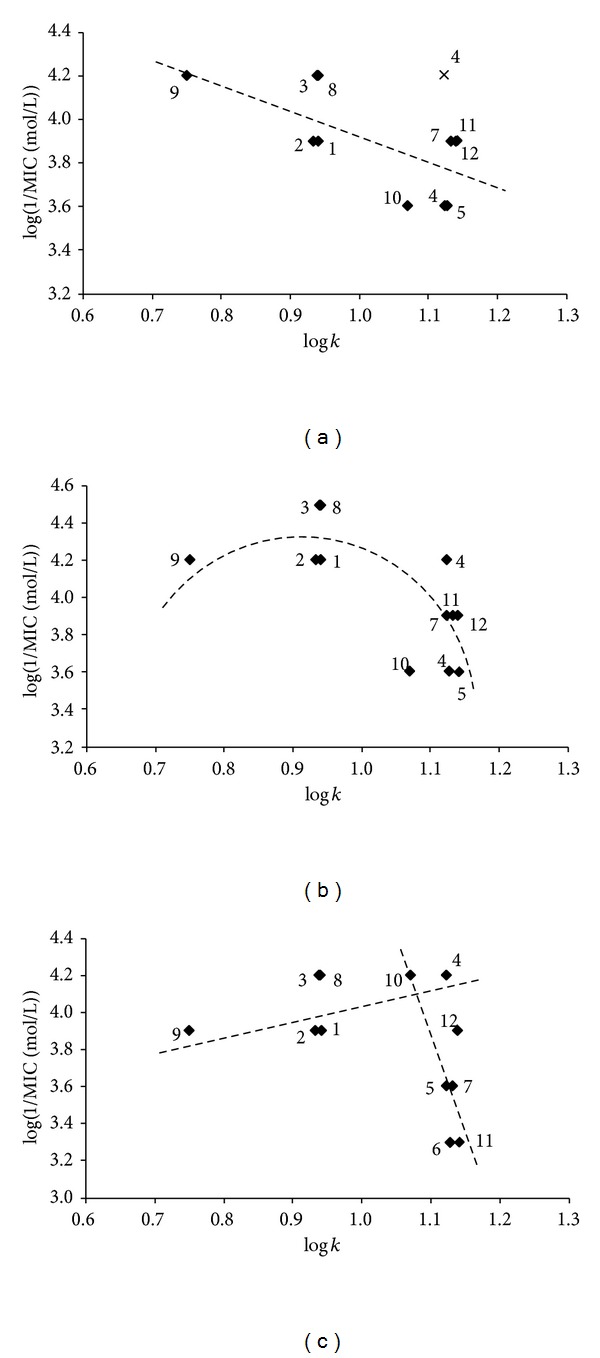
Dependence of* in vitro* antitubercular/antimycobacterial activities against three strains determined after 14 days expressed as log(1/MIC (mol/L)) on lipophilicity expressed as log* k* of 2-[(*E*)-2-substituted-ethenyl]-1,3-benzoxazoles** 1**–**12**:* M. tuberculosis* My 331/88 (a),* M. avium* My 330/88 (b), and* M. kansasii* My 235/80 (c). (Eliminated compound** 4** is marked by cross).

The discussed benzoxazoles** 1**–**12** were tested against three mycobacterial strains:* M. tuberculosis* My 331/88,* M. avium* My 330/88, and* M. kansasii* My 235/80. According to the results of* in vitro* evaluation (see Table 1), none of the compounds showed any activity against* M. tuberculosis*; nevertheless, the activity of some benzoxazole derivatives against* M. kansasii* and especially against* M. avium* considerably exceeded the activity of isoniazid (INH) used as the standard. Compound** 6** had the lowest activity, and 2-[(*E*)-2-(4-methoxyphenyl)ethenyl]-1,3-benzoxazole (**3**) and 2-[(*E*)-2-(2,3-dihydro-1-benzofuran-5-yl)ethenyl]-1,3-benzoxazole (**8**) had the highest antitubercular/antimycobacterial activities against all three strains; see Table 1. It is important to note that 2,3-dihydro-1-benzofuran-5-yl (substituent of 1,3-benzoxazole in compound** 8**) is a cyclic analogue of the 4-methoxyphenyl moiety (compound** 3**), and this group is also isosteric to the methylsulfanyl moiety (compound** 4**), which is another potentially effective substituent. Although the number of compounds demonstrating antimycobacterial activity is limited and the solubility of some compounds in the testing medium was restricted, correlations between log(1/MIC (mol/L)) and lipophilicity expressed as log* k* can be found; see Figure 2. Despite appreciable variance observed in MIC values it can be generally concluded that antitubercular/antimycobacterial activity is especially influenced by lipophilicity. It seems that, with the exception of sulfuric isostere** 4**, the antitubercular activity decreases approximately linearly with increasing lipophilicity (Figure 2(a)). On the other hand, the dependence of log(1/MIC (mol/L)) on log* k* was quasiparabolic for* M. avium *(Figure 2(b)), while for* M. kansasii *(Figure 2(c)) the antimycobacterial activity moderately increased with the increasing lipophilicity of the compounds (log* k* from 0.7503 (**9**) to 1.0702 (**10**)). For compounds with higher lipophilicity (log* k* ranged from 1.1226 (**6**) to 1.1411 (**11**)) the inhibitory activity varied from 62.5 *μ*mol/L (**4**) to 500 *μ*mol/L (**6**,** 11**). However, due to the limited solubility of some compounds in the testing medium considerable variance in MIC values was observed, and clear dependence of log(1/MIC (mol/L)) on log* k* in this range of lipophilicity could not be found.

Also the following SAR observations were made for this series of compounds. Substitution by the phenyl ring showed similar activity as substitution by the furanyl ring. Substitution of* ortho*-position decreased activity in comparison with substitution of* para*-position (see compounds** 2** and** 3**). Substitution of phenyl ring by electron-donor moieties such as the methoxy group (compound** 3**) seems to be more advantageous than substitution by electron-withdrawing, for example, chloro- or trifluoromethyl, moieties (compounds** 6**,** 7**). This observation is different than in case of ring-substituted 2-phenyl-5,7-di-*tert*-butylbenzoxazoles [10]. Prolongation of ethenyl linker to the butadienyl chain decreased activity; compare** 1** and** 11**. Generally it can be concluded that the 4-methoxy moiety is a favourable substituent within the series of compounds; compare** 3** and** 1** or** 12** and** 11**.

#### 3.2.2. Inhibition of Photosynthetic Electron Transport (PET) in Spinach Chloroplasts

As was mentioned above, drugs and agrochemicals as biologically active compounds can target similar sites of action. Thus herbicides can also have molecular sites of action in mammals/nonplant organisms, but targeting compounds to biological systems with similar physicochemical properties can lead to completely different biological responses in plants and animals. For example, fluconazole was firstly discovered as a potent pesticide, and subsequently it was confirmed as an antifungal drug [37]. Moreover, extensive screening of novel compounds is needed and recommended in order to estimate the influence of these compounds on different nontarget organisms after entry into the environment [52, 53].

**Figure 3:**
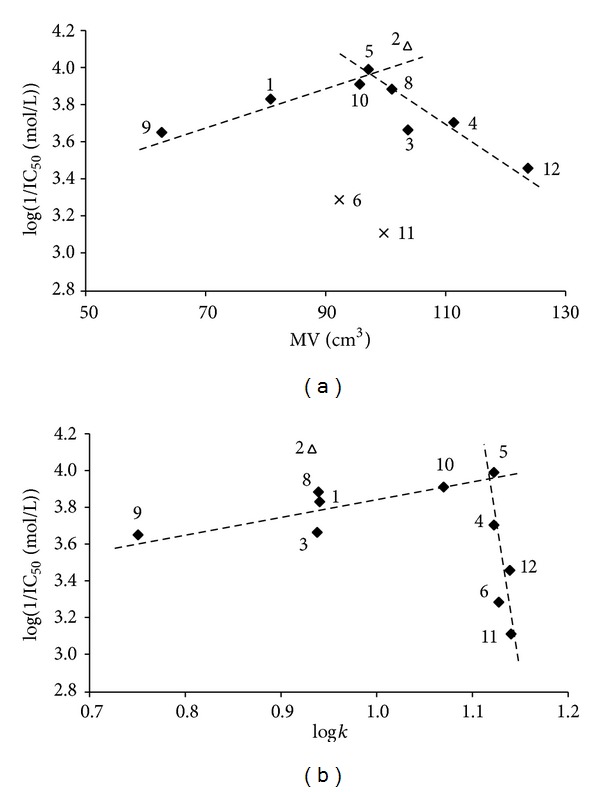
Relationships between PET inhibition log(1/MIC (mol/L)) in spinach chloroplasts and molar volume (MV (cm^3^)) of individual substituents in 2-[(*E*)-ethenyl]-1,3-benzoxazole (a) and lipophilicity expressed as log* k* (b) of selected studied compounds. (Eliminated compounds** 6** and** 11** are marked by cross; the most active* ortho*-substituted compound** 2** is marked by triangle).

The activity of the evaluated substituted benzoxazoles related to inhibition of photosynthetic electron transport (PET) in spinach (*Spinacia oleracea* L.) chloroplasts was moderate or low relative to the standard; see Table 1. The PET-inhibiting activity of compounds was expressed by negative logarithm of IC_50_ value (compound concentration in mol/L causing 50% inhibition of PET) and varied from 76.3 *μ*mol/L (2-[(*E*)-2-(2-methoxyphenyl)ethenyl]-1,3-benzoxazole,** 2**) to 777.5 *μ*mol/L (2-[(1*E*,3*E*)-4-phenylbuta-1,3-dien-1-yl]-1,3-benzoxazole,** 11**). The PET-inhibiting activity of compound** 7** could not be determined due to precipitation of the compounds during the experiments. 2-[(*E*)-2-(2-Methoxyphenyl)ethenyl]-1,3-benzoxazole (**2**), the only* ortho*-substituted compound, showed the highest PET-inhibiting activity (IC_50_ = 76.3 *μ*mol/L). Effective PET inhibition was observed previously also for some ring-substituted salicylanilides and carbamoylphenylcarbamates substituted in position 2 [39]. However, to ascertain whether substitution in position 2 would be advantageous from the aspect of higher PET inhibiting activity also for the studied 2-[(*E*)-2-substituted-ethenyl]-1,3-benzoxazoles, more detailed study with a set of 2-substitued compounds would be necessary. Despite the relatively low inhibitory activity of the studied compounds, correlations between log(1/IC_50_ (mol/L)) and the molar volume (MV (cm^3^)) of individual substituents in C_(2)_ position of the 2-[(*E*)-ethenyl]-1,3-benzoxazole scaffold or the lipophilicity of compounds expressed as log* k* were found; see Figure 3.

Based on the obtained results (see Table 1, Figure 3(a)) it can be stated that PET-inhibiting activity is influenced by the molar volume of substituents. When derivatives** 6** and** 11** showing the lowest PET-inhibiting activity are eliminated, the bilinear dependence of log(1/IC_50_ (mol/L)) on the molar volume can be observed (Figure 3(a)). On the other hand, the biological activity is also affected by lipophilicity. In general, for heterocyclic and/or* para*-substituted derivatives of 2-[(*E*)-ethenyl]-1,3-benzoxazole a linear increase of PET inhibiting activity with increasing lipophilicity to log* k* ca. 1.1 was only moderate, which is presented in Figure 3(b) where the dependence of log(1/IC_50_ (mol/L)) on log* k* is illustrated. However, the subsequent increase of lipophilicity is associated with the rapid decline of PET-inhibiting activity which could be connected with decreasing solubility of the compounds.

An experiment with 2,5-diphenylcarbazide (DPC), an artificial electron donor acting in Z/D intermediate on the donor side of PS II, was performed to specify the site of action of the tested compounds in the photosynthetic apparatus. Practically complete restoration of photochemical activity of chloroplasts (up to 93% of the control) that was previously suppressed by the tested compounds indicated that their site of action is situated on the donor side of PS II. In our previous studies we found that the site of inhibitory action of* N*-substituted 2-aminobenzothiazoles [25], ring-substituted 3-hydroxynaphthalene-2-carboxanilides [42], and 2-hydroxynaphthalene-1-carboxanilides [43] as well as 5-bromo- and 3,5-dibromo-2-hydroxy-*N*-phenylbenzamides [54] is also situated exclusively on the donor side of PS II, and these compounds, similarly to studied 2-[(*E*)-2-substituted-ethenyl]-1,3-benzoxazoles, did not damage the core of PS II (P680).

**Figure 4:**
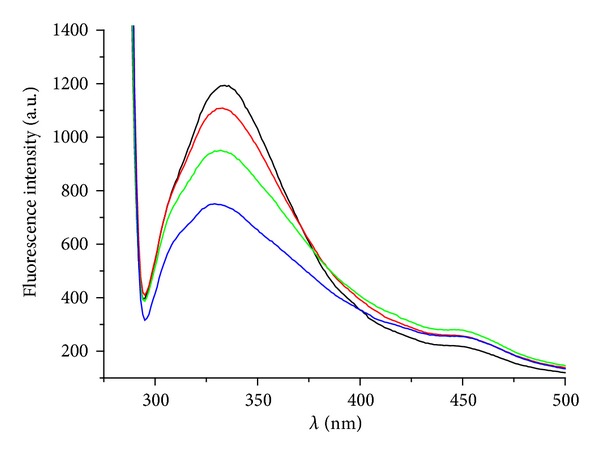
Emission fluorescence spectra of aromatic amino acids in suspension of spinach chloroplasts without and with** 3 **(*c* = 0, 3.2, 6.4, 9.6 *μ*mol/L, curves from top to bottom).

The interaction of studied compounds with aromatic amino acids (AAA) occurring in photosynthetic proteins of spinach chloroplasts situated in PS II was monitored by the quenching of AAA fluorescence at 334 nm. The fluorescence emission spectra of AAA of untreated spinach chloroplasts and of compound** 3** are presented in Figure 4. A strong decline of AAA fluorescence with increasing concentration of** 3** indicates its interaction with these constituents of photosynthetic apparatus resulting ultimately in PET inhibition. Similar interaction with AAA was observed previously for several PET inhibitors, for example, substituted* N-*benzylpyrazine-2-carboxamides [55], ring-substituted 2-hydroxy-naphthalene-1-carboxanilides [43], and 5-bromo- and 3,5-dibromo-2-hydroxy-*N*-phenylbenzamides [54].

## 4. Conclusion

A series of twelve 2-[(*E*)-2-substituted-ethenyl]-1,3-benzoxazoles was prepared and characterized; six derivatives were prepared for the first time. The prepared compounds were tested for their ability to inhibit photosynthetic electron transport (PET) in spinach chloroplasts (*Spinacia oleracea* L.) and for their antitubercular/antimycobacterial activity against* Mycobacterium tuberculosis*,* M. kansasii,* and* M. avium*. 2-[(*E*)-2-(2-Methoxyphenyl)ethenyl]-1,3-benzoxazole (**2**) showed the highest PET inhibition within the whole series of compounds, while the PET-inhibiting activity of* para*-substituted compounds was significantly lower. It was determined that the site of action of the tested compounds is situated on the donor side of photosystem II. 2-[(*E*)-2-(4-Methoxyphenyl)ethenyl]-1,3-benzoxazole (**3**), 2-{(*E*)-2-[4-(methylsulfanyl)phenyl]-ethenyl}-1,3-benzoxazole (**4**), and 2-[(*E*)-2-(2,3-dihydro-1-benzofuran-5-yl)ethenyl]-1,3-benzoxazole (**8**) expressed significantly higher activity against* M. avium* and* M. kansasii* than the standard isoniazid. All three compounds showed comparable activity; compound** 8** is a cyclic analogue of** 3** and compound** 4** is an isostere of** 3**. It can be stated that lipophilicity, type, and position of substitution play the key role in influencing the antimycobacterial activity of compounds.

## Figures and Tables

**Table 1:** Structure of 2-substituted benzoxazoles **1**–**12**, experimentally determined values of lipophilicity log⁡⁡*k* and predicted parameters of individual substituents: distributive parameters *π*, molar volume MV [cm^−3^], and *in vitro* antimycobacterial activities [MIC (*μ*mol/L)] in comparison with standard isoniazid (INH) and IC_50_ [*μ*mol/L] values related to PET inhibition in spinach chloroplasts in comparison with 3-(3,4-dichlorophenyl)-1,1-dimethylurea (DCMU) standard.

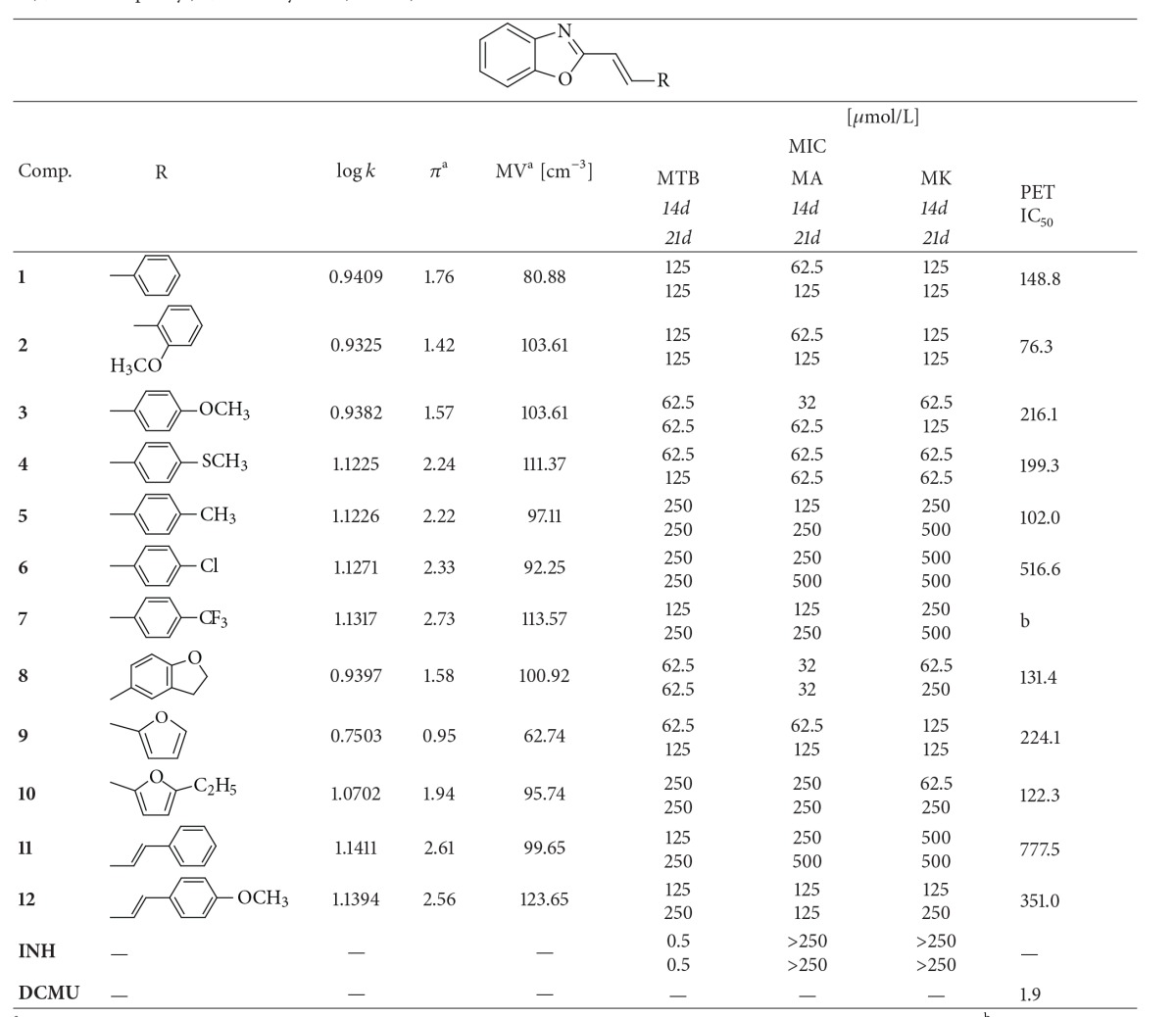

^a^calculated for the uncharged molecules using ACD/Percepta (Advanced Chemistry Development, Inc., Toronto, ON, Canada, 2012); ^b^precipitation during experiment; MTB: *Mycobacterium tuberculosis* My 331/88; MA: *M. avium *My 330/88; and MK: *M. kansasii *My 235/80.
